# Montreal Veterinary College

**Published:** 1884-07

**Authors:** 


### REPORTS.

### MONTREAL VETERINARY COLLEGE.

The final examinations of the students for the Diploma of this school took place on the 27th of last March. The Board was constituted as follows: F. S. Billings, M.V., Roxbury, Mass.; Williamson Byden, V.S., Boston; C. J. Alloway, V.S., Montreal; Archibald McCormick, V.S., Ormstown; Charles Levesque, V.S., Berthie-en-haut; J. A. Couture, V.S., Quebec; George Leclerc, M.D., Montreal; and George O. Beaudry, M.D., Montreal. The following students presented themselves before the Board, having complied with the requirements of the College, attended three full sessions of six months each, and passed the examinations in botany, histology, physics, chemistry, physiology, materia medica, anatomy, practice of medicine and surgery, and general pathology. They were found to be fully competent to practice as veterinary surgeons, and were granted the diploma of the College: Messrs. Ball, Bancroft, Blanchard, Cross, Crundall, Dronin, Duncan, Klock, Labelle, Mears, Morin, and McEachran.

The presentation of prizes won during the session and the diplomas of the College took place in the afternoon at 4 o'clock, when there were present the Rev. Mr. Pilot, Rev. Canon Carmichael, Mr. Casgrain, Mr. Blackwood, and Dr. Leclerc, members of the Council of Agriculture, besides a large number of the friends of the students. S. N. Blackwood, Esq., of Shefford, presided, and on behalf of the Council of Agriculture presented the diplomas and the following prizes :

In the English Class—Seniors—For general proficiency, highest aggregate number of marks in all subjects : prize, a silver medal, the gift of the Council of Agriculture, won by J. A. Duncan.

Pathology and Practice of Veterinary Medicine and Surgery —1st prize, book, won by J. A. Duncan; 2d prize, book, won by W. H. Klock.

Entozoa—Prize won by M. G. Blanchard.

Anatomy—Prize, book, won by Charles McEachran.

Materia Medica—Prize won by E. W. Hoare.

Juniors—Pathology and Practice of Medicine and Surgery—Prize, book, won by E. W. Hoare.

Anatomy—Prize, book, won by C. G. Lamb.

French Class—Best general examinations—Prize, medal, the gift of the Council of Agriculture, won by C. L. Morin.

Pathology—Prize, instrument, won by Jos. Labelle.

Anatomy—Prize, book, won by C. L. Morin.

Juniors—Best general examination—Prize, instrument, won by J. Turcot.

Freshmen—Best general examination—Prize, instrument, won by Louis Lorrain.

The prizes and diplomas having been presented, addresses were delivered by Mr. Billings, Dr. Beaudry, Principal McEachran, and Rev. Canon Carmichael, all of whom gave appropriate advice to the graduating class.

*Montreal Veterinary Medical Association.*

A meeting of this Association was held in the College lecture-room at the close of the public exercises, the President, Mr. M. C. Baker, in the chair.

It was moved by Mr. Magor, seconded by Mr. Lamb, and resolved, that Messrs. Ball, Blanchard, Cross, Crundall, Mears, McEachran, Dronin, Labelle, and Morin, having complied with the requirements, be granted the diploma of honorary fellowship of the Association, which was accordingly done.

After a few remarks from the President, the meeting adjourned until the first Thursday of October next.

*The Annual Dinner.*

The annual dinner of the students of the Veterinary College was held at the St. Lawrence Hall, March 27th. The chair was occupied by Prof. D. McEachran, Principal of the College, who had on his right and left Mr. Billings, Dr. Leclerc, Dr. Beaudry, B. J. Coghlin, W. Stephens, and G. Shewan. Among the many others present were Drs. Bell, F. W. Campbell, Ross, Buller, and Stewart, Prof. Penhallow, Mr. C. Alloway, and Mr. Baker, V.S.

